# Novel hybrid visual stimuli incorporating periodic motions into conventional flickering or pattern-reversal visual stimuli for steady-state visual evoked potential-based brain-computer interfaces

**DOI:** 10.3389/fninf.2022.997068

**Published:** 2022-09-21

**Authors:** Jinuk Kwon, Jihun Hwang, Hyerin Nam, Chang-Hwan Im

**Affiliations:** ^1^Department of Biomedical Engineering, Hanyang University, Seoul, South Korea; ^2^Department of Electronic Engineering, Hanyang University, Seoul, South Korea; ^3^Department of Artificial Intelligence, Hanyang University, Seoul, South Korea; ^4^Department of HY-KIST Bio-Convergence, Hanyang University, Seoul, South Korea

**Keywords:** brain-computer interfaces (BCIs), steady-state visual evoked potential (SSVEP), steady-state motion visual evoked potential (SSMVEP), hybrid visual stimulus, periodic motion

## Abstract

In this study, we proposed a new type of hybrid visual stimuli for steady-state visual evoked potential (SSVEP)-based brain-computer interfaces (BCIs), which incorporate various periodic motions into conventional flickering stimuli (FS) or pattern reversal stimuli (PRS). Furthermore, we investigated optimal periodic motions for each FS and PRS to enhance the performance of SSVEP-based BCIs. Periodic motions were implemented by changing the size of the stimulus according to four different temporal functions denoted by none, square, triangular, and sine, yielding a total of eight hybrid visual stimuli. Additionally, we developed the extended version of filter bank canonical correlation analysis (FBCCA), which is a state-of-the-art training-free classification algorithm for SSVEP-based BCIs, to enhance the classification accuracy for PRS-based hybrid visual stimuli. Twenty healthy individuals participated in the SSVEP-based BCI experiment to discriminate four visual stimuli with different frequencies. An average classification accuracy and information transfer rate (ITR) were evaluated to compare the performances of SSVEP-based BCIs for different hybrid visual stimuli. Additionally, the user's visual fatigue for each of the hybrid visual stimuli was also evaluated. As the result, for FS, the highest performances were reported when the periodic motion of the sine waveform was incorporated for all window sizes except for 3 s. For PRS, the periodic motion of the square waveform showed the highest classification accuracies for all tested window sizes. A significant statistical difference in the performance between the two best stimuli was not observed. The averaged fatigue scores were reported to be 5.3 ± 2.05 and 4.05 ± 1.28 for FS with sine-wave periodic motion and PRS with square-wave periodic motion, respectively. Consequently, our results demonstrated that FS with sine-wave periodic motion and PRS with square-wave periodic motion could effectively improve the BCI performances compared to conventional FS and PRS. In addition, thanks to its low visual fatigue, PRS with square-wave periodic motion can be regarded as the most appropriate visual stimulus for the long-term use of SSVEP-based BCIs, particularly for window sizes equal to or larger than 2 s.

## Introduction

Brain-computer interfaces (BCIs) are promising alternative communication technologies that have been generally developed for people who suffer from neuromuscular disorders or physical disabilities such as spinal cord injury, amyotrophic lateral sclerosis, and locked-in syndrome (Daly and Wolpaw, 2008). BCIs have provided new non-muscular communication channels that allowed for interaction between a user and the external environment. A variety of non-invasive brain imaging modalities have been employed to record brain activities in the field of BCIs. For example, functional magnetic resonance imaging (fMRI), magnetoencephalography, and functional near-infrared spectroscopy have been successfully employed to implement BCIs. In addition, electroencephalography (EEG) is another representative non-invasive neuroimaging modality that has been the most intensively studied owing to its advantages over the other modalities, such as high temporal resolution, affordability, and portability (Dai et al., 2020; Zhang et al., 2021).

In the EEG-based BCIs, the user performs certain mental tasks according to paradigms designed for eliciting task-related neural activities. Motor imagery, event-related potential, P300, and auditory steady-state response are popular paradigms employed to implement EEG-based BCIs (Lotte et al., 2018; Abiri et al., 2019). Steady-state visual evoked potential (SSVEP) is also one of the most promising EEG-based BCI paradigms, which has attracted increased interest from BCI researchers in recent decades (Waytowich et al., 2018). SSVEPs are periodic brain activities evoked in response to the presentation of visual stimulus flickering or pattern-reversing at a specific temporal frequency. SSVEP signals are entrained at the fundamental and harmonic frequencies of the visual stimulus and are well-known to be mainly observed in the occipital region of the brain over a wide range of 1–90 Hz (Herrmann, 2001; Choi et al., 2019a). SSVEP-based BCIs interpret the user's intention by detecting the visual stimulus that the user gazed at based on these characteristics and have various advantages over the other paradigms, such as high information transfer rate (ITR), excellent stability, and little training requirement (Zhang et al., 2020; Kim and Im, 2021). Thanks to these advantages, SSVEP-based BCIs have been successfully applied to various applications including mental speller (Nakanishi et al., 2018), assistive technology for patients (Perera et al., 2016), online home appliance control (Kim et al., 2019), and hands-free controllers for virtual reality (VR) (Armengol-Urpi and Sarma, 2018) or augmented reality (AR) (Arpaia et al., 2021).

In general, two types of visual stimuli have been employed to evoke SSVEPs: (1) flickering stimulus (FS) and (2) pattern-reversal stimulus (PRS) (Bieger et al., 2010; Zhu et al., 2010). FS is the visual stimulus that modulates the color or luminance of the stimulus at a specific frequency. Flickering single graphics in the form of squares or circles rendered on an LCD monitor is the representative FS used to elicit the SSVEPs. PRS evokes SSVEP responses by alternating the patterns of the visual stimuli (e.g., checkerboard or line boxes) at a constant frequency. Based on these visual stimuli, a number of studies have been conducted to improve the performance of SSVEP-based BCIs, examples of which include optimization of stimulus parameters such as spatial frequency of PRS, stimulation frequencies, colors, and waveform of FS (Bieger et al., 2010; Teng et al., 2011; Duszyk et al., 2014; Jukiewicz and Cysewska-Sobusiak, 2016; Chen et al., 2019). Recently, Choi et al. (2019b) and Park et al. (2019) proposed a novel type of visual stimulus called grow/shrink stimulus (GSS) to improve the performance of SSVEP-based BCI in AR and VR environments, respectively. GSS was implemented by incorporating a periodic motion into FS to concurrently evoke SSVEP and steady-state motion visual evoked potential (SSMVEP), inspired by previous studies that reported that the periodic motion-based visual stimuli could elicit SSMVEP (Xie et al., 2012; Yan et al., 2017). GSS has shown a higher BCI performance compared to the conventional PRS or FS in both VR and AR environments. However, no study has been conducted on the performance of GSS-like visual stimuli for SSVEP-based BCI when the LCD monitor is used as a rendering device. Furthermore, the effect of the motion parameters (i.e., the waveform of the temporal motion dynamics) on the BCI performances has not been investigated. Indeed, the investigation of the performance of various GSS-like visual stimuli with the LCD monitor environment is important because most SSVEP-based BCI studies employ the LCD monitor to present the visual stimuli (Ge et al., 2019; Chen et al., 2021; Xu et al., 2021). In addition, to the best of our knowledge, hybrid visual stimuli that consolidate PRS with periodic motions have never been proposed in previous studies.

In this study, we proposed novel hybrid visual stimuli that consolidate the conventional PRS with periodic motions and further investigated the effect of waveforms of the periodic motions for hybrid visual stimuli based on either FS or PRS on the performance of SSVEP-based BCIs. As for the periodic motions, the stimulus size was changed according to four different waveforms: none (no change in the size), square (changing size in a binary manner), triangular (linearly increasing and decreasing size), and sine (changing size with a sinusoidal waveform), resulting in a total of eight different hybrid visual stimuli (i.e., FS and PRS each with four periodic motions). We evaluated two crucial factors for the practical use of SSVEP-based BCIs: (1) BCI performances and (2) visual fatigue, for each visual stimulus, with 20 healthy participants. A filter bank canonical correlation analysis (FBCCA) algorithm, which is a state-of-the-art training-free algorithm for SSVEP-based BCIs was employed to evaluate the performances of SSVEP-based BCIs in terms of classification accuracy and information transfer rate (ITR). Moreover, an extended version of FBCCA, named subharmonic-FBCCA (sFBCCA) was developed for the SSVEP-based BCIs with PRS-based hybrid visual stimuli.

## Methods

### Participants

A total of 20 healthy adults (10 males, aged 23.7 ± 3.5 years) with normal or corrected-to-normal vision participated in the experiments. None of the reported any serious history of neurological, psychiatric, or other severe diseases that could otherwise influence the experimental results. All participants were informed of the detailed experimental procedure and provided written consent before the experiment. This study and the experimental paradigm were approved by the Institutional Review Board Committee of Hanyang University, Republic of Korea (IRB No. HYU-202006-004-03) according to the Declaration of Helsinki.

### Visual stimuli

**Figure 1:**
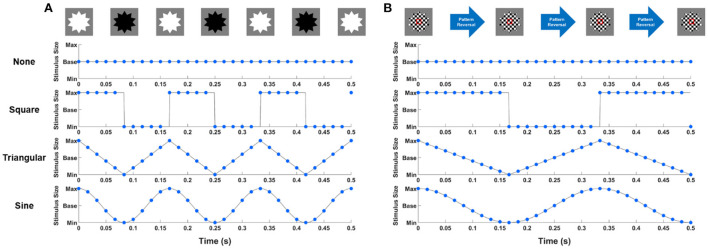
Examples of **(A)** FS-based hybrid visual stimuli and **(B)** PRS-based hybrid visual stimuli when the stimulation frequency was 6 Hz. Blue circles indicate the stimulus size presented to participants considering the refresh rate of the LCD monitor.

The visual stimuli were developed with the Unity 3D engine (Unity Technologies ApS, San Francisco, CA, USA). Based on previous GSS studies (Choi et al., 2019b; Park et al., 2019), all stimuli were designed in a star shape, with a base size of 7 cm (5.7°) to increase the visibility of periodic motions. The background color was set to gray. Both FS and PRS changed the color or reversed the patterns with the periodic square waveform according to the results of previous studies that reported that square-wave FS exhibited significantly higher classification accuracy than FS of other waveforms (Teng et al., 2011; Chen et al., 2019). The periodic motions were implemented by varying the size of visual stimuli according to four different types of waveforms: none (no change in the size), square (changing size in a binary manner), triangular (linearly increasing and decreasing size), and sine (changing size with a sinusoidal waveform) waveforms with a modulation ratio of 33% compared to the base size (i.e., the radius of each stimulus was changed from 0.67 to 1.33 when the radius of the base stimulus was assumed to be one). The conventional visual stimuli of FS and PRS were combined with four periodic motions, resulting in eight hybrid visual stimuli. Hereinafter, none, square, triangular, and sine waveforms are referred to as None, Square, Triangular, and Sine, respectively, and each hybrid visual stimuli are referred to as FS-None, FS-Square, FS-Triangular, FS-Sine, PRS-None, PRS-Square, PRS-Triangular, and PRS-Sine. Note that FS-None and PRS-None were the same as the conventional FS and PRS with the base size. Figure 1 illustrates the examples of the hybrid visual stimuli when the stimulation frequency was set to 6 Hz. Blue circles indicate the stimulus size presented to the participants considering the refresh rate of the LCD monitor (= 60 Hz). It is worthwhile noting that the most important difference between FS and PRS is that FS elicits SSVEP responses at the number of full cycles (i.e., two reversals) per second, whereas PRS evokes SSVEP responses at the number of reversals per second (Zhu et al., 2010). Therefore, the stimulation frequencies of periodic motions for PRS were set to be half of those for FS, which were considered as subharmonics of the stimulation frequencies in the further analysis.

### Experimental paradigm

**Figure 2:**
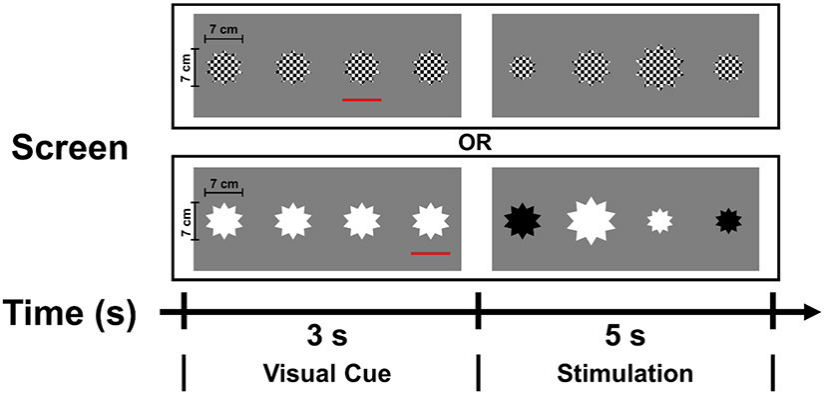
The timing sequence of a single trial. Each trial consisted of the visual cue of 3 s and the stimulation time of 5 s.

The participants sat 70 cm away from a 27-inch LCD monitor with a resolution of 1920 x 1080 pixels and the 60 Hz refresh rate. The experiment consisted of eight sessions corresponding to each hybrid visual stimuli and the order of the sessions was randomized for each participant. Each session was composed of 20 trials (5 trials × 4 stimuli), each of which consisted of the visual cue of 3 s and the stimulation time of 5 s. The red bar was presented under the target stimulus during visual cue period in a randomized order. The timing sequence of a single trial is shown in Figure 2. The stimulation frequencies of four stimuli were determined as 6, 6.67, 7.5, and 10 Hz considering the refresh rate of the LCD monitor. In each trial, the participants were instructed to focus their attention on the target stimulus among four simultaneously flickering stimuli without eye blinks and body movements during the stimulation time. At the end of each session, the participants evaluated the visual fatigue score for each hybrid visual stimulus in the range of 1–10 (1, low fatigue; 10, high fatigue).

### Data recording and pre-processing

EEG data were recorded from eight scalp electrodes (O1, Oz, O2, PO7, PO3, POz, PO4, and PO8) using a commercial EEG system (BioSemi Active Two; Biosemi, Amsterdam, The Netherlands) at a sampling rate of 2,048 Hz. A CMS active electrode and a DRL passive electrode were used to form a feedback loop for the amplifier reference (Park et al., 2019). MATLAB 2020b (Mathworks; Natick, MA) was used to analyze the EEG data, and the functions implemented in the BBCI toolbox (https://github.com/bbci/bbci_public) were employed. The raw EEG data were down-sampled to 256 Hz to reduce the computational cost and then bandpass-filtered using a sixth-order zero-phase Butterworth filter with cutoff frequencies of 2 and 54 Hz. Considering a latency delay in the visual pathway, the EEG data were segmented into epochs from 0.135 to 0.135+*w* s with respect to the task onset time (0 s), where *w* indicates the window size used for SSVEP detection (Rabiul Islam et al., 2017).

### Classification methods

#### Canonical correlation analysis

CCA is a multivariate statistical method used to measure the underlying correlation between two sets of multidimensional variables, $X\in R^{d_{x}\times N_{s}}$ and $Y\in R^{d_{y}\times N_{s}}$ where, *N*_*s*_ is the number of sample points and *d*_*x*_ and *d*_*y*_ indicate the dimension of *X* and *Y*, respectively (Nakanishi et al., 2015). Considering their linear combinations $x=\;X^{T}W_{X}$ and $y=\;Y^{T}W_{Y}$, CCA finds a pair of weight vectors $W_{X}\in R^{d_{x}\times 1}$ and $W_{Y}\in R^{d_{y}\times 1}$ that maximize Pearson's correlation coefficients between *x* and *y* using the following equation:


(1) $$\begin{array}{r} \underset{W_{x},W_{y}}{\max}\rho \left(x,y\right)=\;\frac{E\left[W_{X}^{T}XY^{T}W_{Y}\right]}{\sqrt{E\left[W_{X}^{T}XX^{T}W_{X}\right]E\left[W_{Y}^{T}YY^{T}W_{Y}\right]}}\;. \end{array}$$

Here, *T* denotes the transpose operation. The maximum correlation coefficient with respect to *W*_*X*_ and *W*_*Y*_ is called the “CCA coefficient.”

For SSVEP detection, the CCA coefficients, ρ_*f*_, between multichannel EEG signals, $X\in R^{N_{c}\times N_{s}}$, and the reference signals for each stimulus frequency, $Y_{f}\in R^{{2N}_{h}\times N_{s}}$, were evaluated and the frequency with the largest CCA coefficient was classified as the target frequency, as follows:


(2) $$\begin{array}{r} f_{target}\;=\underset{f}{\max}\rho_{f},\;f=f_{1},f_{2},\ldots ,f_{K}\;. \end{array}$$

Here, *K* is the number of stimulus frequencies presented to the participants.

The reference signal for each stimulus frequency (*Y*_*f*_) was set as


(3) $$Y_{f}=\left[\begin{array}{c} \sin\left(2\pi fn\right)\\ \cos\left(2\pi fn\right)\\ \vdots \\ \sin\left(2\pi N_{h}fn\right)\\ \cos\left(2\pi N_{h}fn\right) \end{array}\right],\;n=\frac{1}{f_{s}},\frac{2}{f_{s}},\ldots ,\frac{N_{s}}{f_{s}}\;,$$

where, *f* is the stimulus frequency. In this study, *N*_*c*_ and *f*_*s*_ denote the number of channels and sampling frequency, which were set to 8 and 256, respectively. *N*_*h*_ represents the number of harmonics, which was set to 5 according to previous studies (Chen et al., 2015).

#### Filter bank CCA

FBCCA combines CCA with filter bank analysis to extract the discriminative information in the harmonic components (Chen et al., 2015). The filter bank is applied to decompose EEG data into multiple sub-band data, and CCA coefficients are evaluated for each sub-band. The weighted sums of the squared sub-band CCA coefficients for each stimulus frequency are calculated using the following equations:


(4) $$\begin{array}{rc} \rho_{f} & =\overset{N_{m}}{\underset{m=1}{\sum }}w\left(m\right)\cdot {\left(\rho_{f}^{m}\right)}^{2}\;, \end{array}$$

(5) $$\begin{array}{rc} w\left(m\right) & =m^{-a}+b\;, \end{array}$$

where, *N*_*m*_ is the number of subbands, *m* is the index of the subbands, and $\rho_{f}^{m}$ denotes the CCA coefficient of sub-band *m*. The target frequency is determined in the same manner as in CCA. According to previous studies (Chen et al., 2015; Zhao et al., 2020), the following parameters were set: *a* = 1.25, *b* = 0.25, and *N*_*m*_ = 5. The filter bank for five sub-bands was designed with lower and upper cutoff frequencies of 4–52, 8–52, 12–52, 16–52, and 20–52 Hz, respectively (Chen et al., 2015). In this study, FBCCA was employed to identify SSVEPs because it is generally regarded as the best available algorithm, yielding the highest classification accuracy without the need for training sessions (Zerafa et al., 2018; Liu et al., 2020).

#### Subharmonic FBCCA (sFBCCA)

In this study, we proposed an extended version of FBCCA, named subharmonic-FBCCA (sFBCCA), to utilize the information in the subharmonic component, elicited by periodic motions for PRS. In sFBCCA, the reference signal was expanded to include the subharmonic component as follows:


(6) $$Y_{f}=\left[\begin{array}{c} \sin\left(\pi fn\right)\\ \cos\left(\pi fn\right)\\ \sin\left(2\pi fn\right)\\ \cos\left(2\pi fn\right)\\ \vdots \\ \sin\left(2\pi N_{h}fn\right)\\ \cos\left(2\pi N_{h}fn\right) \end{array}\right],\;n=\frac{1}{f_{s}},\frac{2}{f_{s}},\ldots ,\frac{N_{s}}{f_{s}}\;.$$

In addition, the equation for the weighted sums of the squared sub-band CCA coefficients is extended as the following equations:


(7) $$\begin{array}{rc} \rho_{f} & =\overset{N_{m}}{\underset{m=1}{\sum }}w\left(m\right)\cdot {\left(\rho_{f}^{m}\right)}^{2}+w_{sub}\cdot {\left(\rho_{f_{sub}}\right)}^{2}, \end{array}$$

(8) $$\begin{array}{rc} w\left(m\right) & =m^{-a}+b, \end{array}$$

(9) $$\begin{array}{rc} w_{sub} & ={m_{sub}}^{-a}+b, \end{array}$$

where, *m*_*sub*_ represents the index of the subharmonic, set to 0.5, in this study. The bandpass filter for the subharmonic component was designed with lower and upper cutoff frequencies of 1–52 Hz. sFBCCA was employed to classify SSVEPs for hybrid visual stimuli of PRS-Square, PRS-Triangular, and PRS-Sine.

### Information transfer rate

In addition to the classification accuracy, ITR (bits per minute) has been widely employed as a metric to assess the performance of the BCI system (Wolpaw et al., 2002). The ITR was evaluated using the following equation:


(10) $$\begin{array}{r} ITR=\frac{60}{T}\left\{log_{p}2N+plog_{p}2p+\left(1-p\right)log_{p}2\left(\frac{1-p}{N-1}\right)\right\}, \end{array}$$

Where, *T* denotes the window size (in seconds), *N* indicates the number of classes, and *p* represents the classification accuracy. In the present study, the *N* value was 4.

### Statistical analysis

Statistical analyses were also performed using MATLAB 2020b (MathWorks; Natick, MA, USA). The non-parametric method was employed because the normality criterion was not satisfied owing to the small sample size. Friedman test was conducted to verify if there were significant differences among the BCI performances. Wilcoxon signed-rank test with the false discovery rates (FDRs) correction for multiple comparisons was performed for *post-hoc* analyses.

## Results

### FS-based hybrid visual stimuli

**Figure 3:**
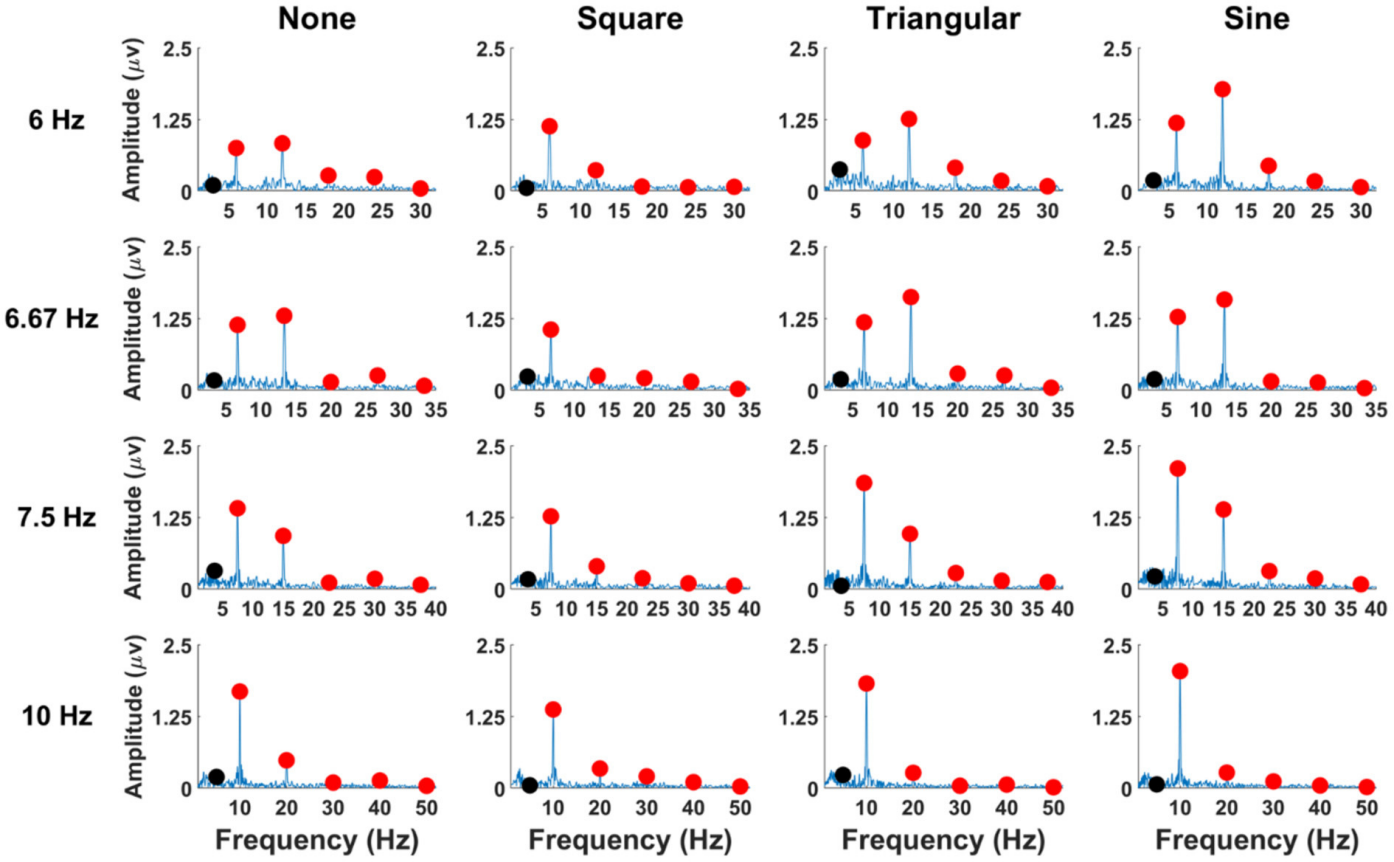
SSVEP amplitudes of the averaged EEG signals across all the participants at the Oz electrode for FS-based hybrid visual stimuli. Red circles indicate the stimulation frequencies and their harmonics, and black circles represent the subharmonic frequencies.

Figure 3 illustrates the grand mean amplitude spectra of SSVEPs averaged across all participants with respect to waveforms of periodic motions. The amplitudes of SSVEPs were obtained from the EEG signals of 5-s long recorded at the Oz electrode. Here, the first five harmonic components of the stimulation frequencies, which were used for the classification, and the subharmonic components were presented in the figure. The red circles indicate the fundamentals and harmonics of stimulation frequencies, and the black circles represent the subharmonics. For FS-based hybrid visual stimuli, clear SSVEP peaks were mainly evoked at the fundamental and second harmonic frequencies. No SSVEP peaks were observed at the subharmonic frequency. The grand average amplitudes of each SSVEP component are listed in Supplementary Table 1.

**Figure 4:**
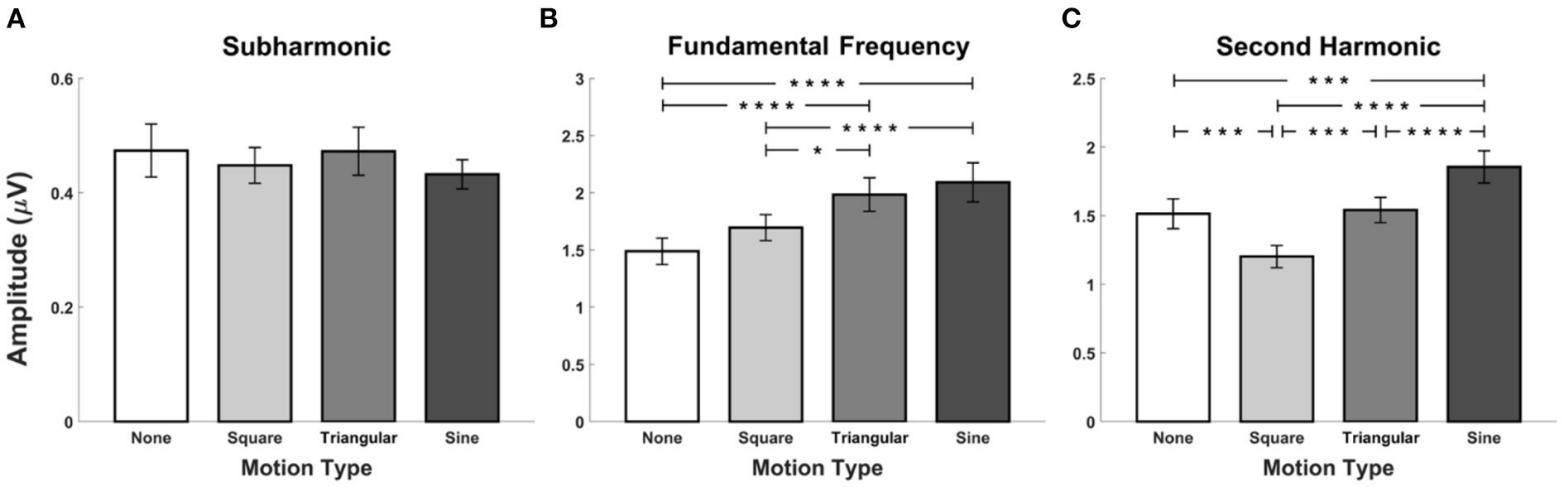
Grand mean SSVEP amplitudes for FS-based hybrid visual stimuli averaged across all the participants at **(A)** the subharmonic frequency, **(B)** the fundamental frequency, and **(C)** the second harmonic frequency. Error bars represent the standard errors. Here, the asterisks of *, ***, and **** represent FDRs-corrected *p* < 0.05, *p* < 0.005, and *p* < 0.001, respectively (Wilcoxon signed-rank test).

The grand mean amplitudes of SSVEP components averaged over all stimulation frequencies across all the participants are illustrated in Figure 4 as a function of waveforms of periodic motions, where the error bars represent the standard errors. The statistical analyses were performed to compare the differences in the amplitude of SSVEP components at the subharmonic, fundamental, and second harmonic frequencies among FS-based hybrid visual stimuli. Four SSVEP amplitudes at each harmonic frequency were calculated from the EEG signals averaged over each stimulation frequency recorded at the Oz electrode for each participant. Consequently, a total of 80 SSVEP amplitudes (4 stimulation frequencies ×20 participants) were statistically compared. The Friedman test indicated significant differences in the amplitudes at fundamental and second harmonic frequencies (subharmonic frequency: χ^2^ = 0.05, *p* = 0.998, fundamental frequency: χ^2^ = 28.26, *p* < 0.001, second harmonic frequency: χ^2^ = 32.81, *p* < 0.001). At the fundamental frequency, SSVEP amplitude elicited by FS-Sine and FS-Triangular was significantly higher than that elicited by FS-None and FS-Square (FDRs-corrected *p* < 0.05 between FS-Square vs FS-Triangular, and FDRs-corrected *p* < 0.001 for the others, Wilcoxon signed-rank test). For the second harmonic frequency, SSVEP amplitude evoked by FS-Sine was significantly higher than that evoked by FS with other waveforms (*p* < 0.005 for FS-None and *p* < 0.001 for the others, Wilcoxon signed-rank test with FDRs correction). Additionally, FS-Square elicited significantly lower SSVEP amplitude than that elicited by the other waveforms (FDRs-corrected *p* < 0.005 for FS-None and FS-Triangular, and FDRs-corrected *p* < 0.001 for FS-Sine, Wilcoxon signed-rank test).

**Figure 5:**
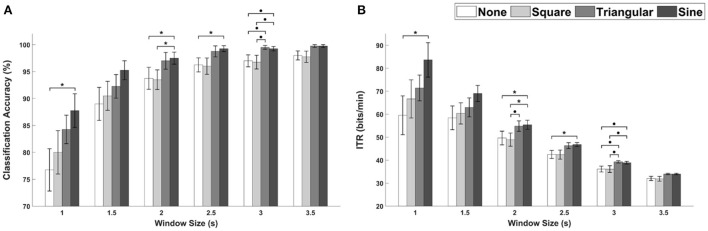
The average performance of FS-based hybrid visual stimuli in terms of **(A)** classification accuracies and **(B)** ITRs as a function of waveforms with different window sizes. Error bars represent the standard errors. Here, ∙*p* < 0.1 and *p < 0.05 are the FDRs-corrected *p-*values from Wilcoxon signed-rank test.

The average classification accuracies and ITRs for FS-based hybrid visual stimuli with respect to different window sizes are depicted in Figures 5A,B, respectively. The Friedman test indicated statistically significant differences for all window sizes except for 1.5 and 3.5 s (1 s, χ^2^ = 8.10, *p* < 0.5; 1.5 s, χ^2^ = 6.20, *p* = 0.102; 2 s, χ^2^ = 9.50, *p* < 0.05; 2.5 s, χ^2^ = 11.38, *p* < 0.001; 3 s, χ^2^ = 9.88, *p* < 0.05; 3.5 s, χ^2^ = 7.47, *p* = 0.058, identical to both the classification accuracy and ITR). The Wilcoxon signed-rank test with FDRs correction showed statistically significant differences in both classification accuracy and ITR between FS-None and FS-Sine for window sizes of 1, 2, and 2.5 s (*p* < 0.5 for both classification accuracies and ITRs). Additionally, the performances of FS-Sine were significantly higher than those of FS-Square for the window size of 2 s (*p* < 0.5, Wilcoxon signed-rank test with FDRs correction). As illustrated in the figure, the performances of SSVEP-based BCI could be improved by incorporating triangular- and sine-wave periodic motions into the conventional FS for all window sizes. For FS, FS-Sine exhibited the highest average performances for every window size except for 3 s, especially for short window sizes.

**Figure 6:**
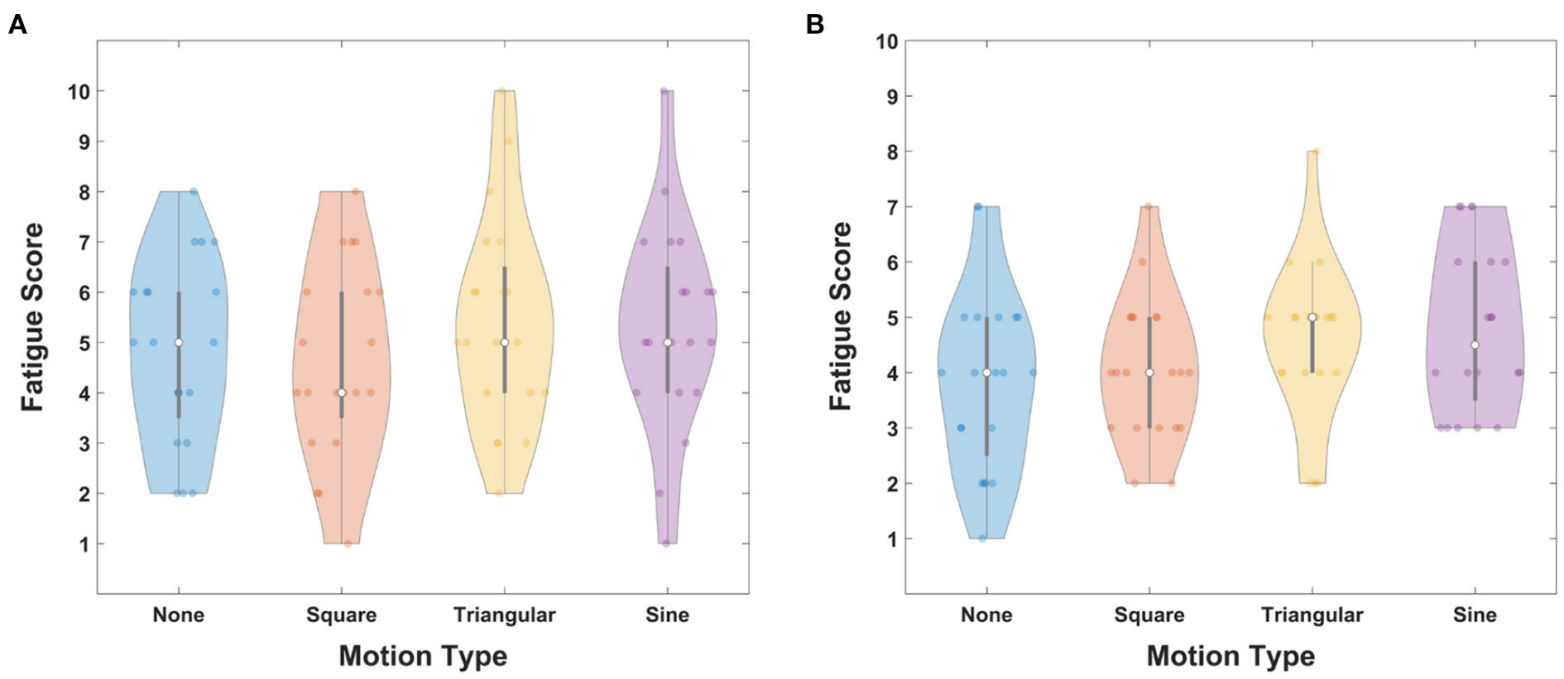
Fatigue scores for **(A)** FS-based hybrid visual stimuli and **(B)** PRS-based hybrid visual stimuli as a function of waveforms. Gray bars represent the interquartile range from 25 to 75% and white circles indicate median values.

Figure 6A illustrates the fatigue scores of FS-based hybrid stimuli as a function of periodic motion waveforms. The gray bars represent the interquartile ranges from the first quartile to the third quartile and white circles indicate the median values. The averaged fatigue scores were 4.8 ± 1.82, 4.6 ± 1.90, 5.4 ± 2.01, and 5.3 ± 2.05 for FS-None, FS-Square, FS-Triangular, and FS-Sine, respectively. A statistically significant difference was not observed in the Friedman test (χ^2^ = 2.55, *p* = 0.467).

### PRS-based hybrid visual stimuli

**Figure 7:**
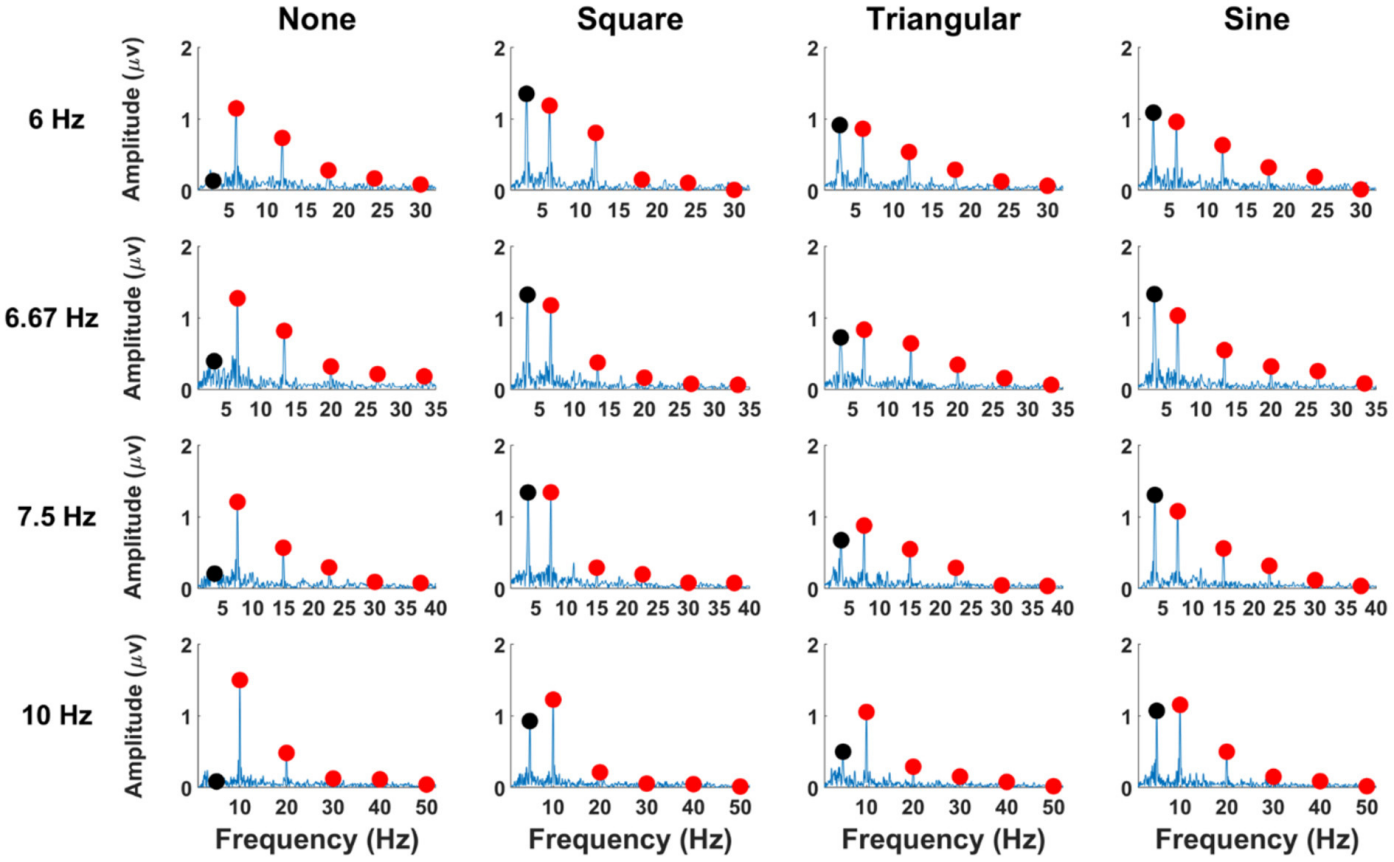
SSVEP amplitudes of averaged EEG signals over all participants for PRS-based hybrid visual stimuli at the Oz electrode. Red circles indicate the stimulation frequencies and their harmonics, and black circles represent the subharmonic frequencies.

The grand mean amplitude spectra of SSVEPs averaged across all participants are illustrated in Figure 7 as a function of waveforms of PRS-based hybrid visual stimuli. The red circles indicate the fundamental and harmonic frequencies, and the black circles represent the subharmonic frequencies. Unlike the FS-based hybrid visual stimuli, clear SSVEP peaks were observed at the subharmonic frequency for PRS-Square, PRS-Triangular, and PRS-Sine, as expected. The grand mean amplitudes of each SSVEP component are listed in Supplementary Table 2.

**Figure 8:**
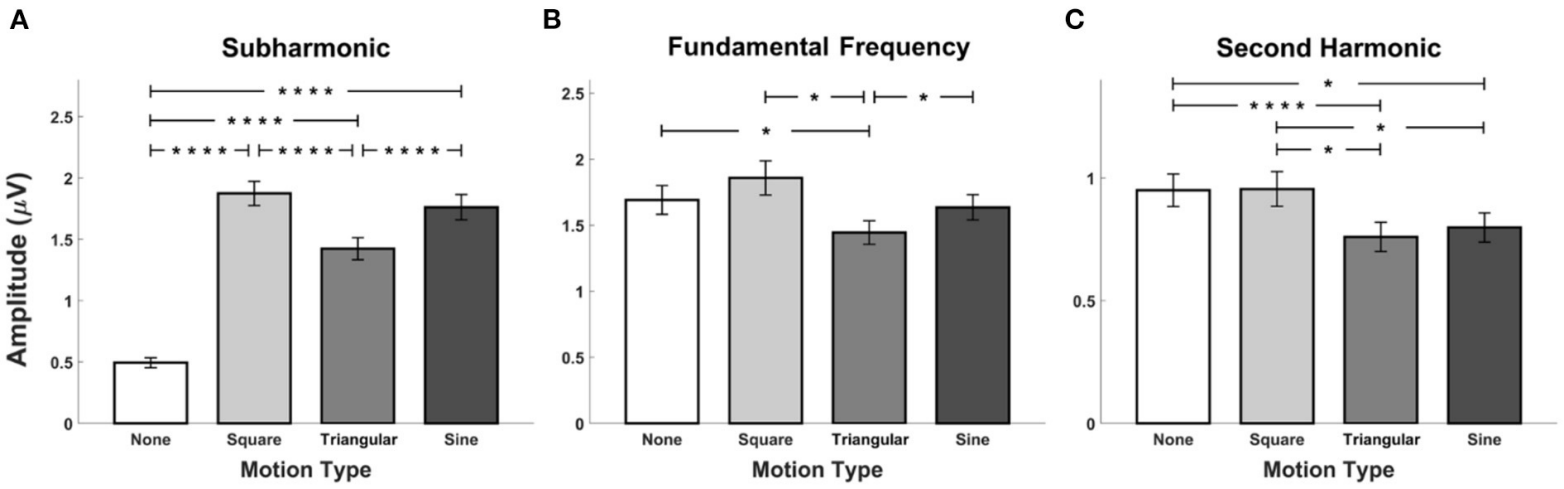
Grand mean SSVEP amplitudes for PRS-based hybrid visual stimuli averaged across all the participants at **(A)** the subharmonic frequency, **(B)** the fundamental frequency, and **(C)** the second harmonic frequency. Error bars represent the standard errors. Asterisks of * and **** represent FDRs-corrected *p* < 0.05 and *p* < 0.001, respectively (Wilcoxon signed-rank test).

The grand mean amplitudes of SSVEP components at the subharmonic, fundamental, and second harmonic frequencies averaged over all stimulation frequencies across all the participants are illustrated in Figure 8 with respect to the periodic motion waveforms incorporated with PRS. The error bars represent the standard errors. The Friedman test indicated significant differences in amplitudes at all harmonic frequencies (χ^2^ = 110.53, *p* < 0.001; χ^2^ = 10.69, *p* < 0.05; χ^2^ = 32.81, *p* < 0.05). PRS-None evoked the lowest SSVEP amplitudes at the subharmonic frequency (*p* < 0.001 for PRS-Square, PRS-Triangular, and PRS-Sine, Wilcoxon signed-rank test with FDRs correction). In addition, the SSVEP amplitude induced by PRS-Triangular was significantly lower than that induced by PRS-Square and Sine (FDRs-corrected *p* < 0.001, Wilcoxon signed-rank test). For the fundamental frequency, PRS-Triangular induced significantly lower SSVEP amplitudes compared to other PRS-based hybrid visual stimuli and even conventional PRS (*p* < 0.05, Wilcoxon signed-rank test with FDRs correction). The SSVEP amplitudes elicited by PRS-None and PRS-Square were significantly higher than those elicited by PRS-Triangular and PRS-Sine at the second harmonic frequency (*p* < 0.001 between PRS-None and PRS-Triangular, and *p* < 0.05 for the others, Wilcoxon signed-rank test, FDRs-corrected).

**Figure 9:**
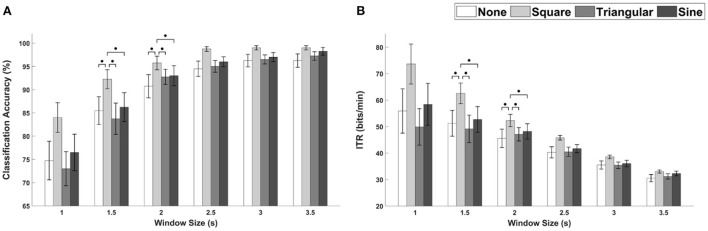
The average performance of PRS-based hybrid visual stimuli in terms of **(A)** classification accuracies and **(B)** ITRs as a function of waveforms with different window sizes. Error bars represent the standard errors. Here, ∙*p* < 0.1 is the FDRs-corrected *p*-value from Wilcoxon signed-rank test.

Figures 9A,B depict the average classification accuracies and ITRs, respectively, for PRS-based hybrid visual stimuli with respect to different window sizes. Here, all the performances of SSVEP-based BCIs were evaluated using FBCCA for PRS-None and sFBCCA for PRS-Square, PRS-Triangular, and PRS-Sine cases. The Friedman test indicated statistically significant differences for window sizes of 1.5 and 2 s (1 s, χ^2^ = 5.59, *p* < 0.133; 1.5 s, χ^2^ = 9.30, *p* < 0.05; 2 s, χ^2^ = 8.06, *p* < 0.05; 2.5 s, χ^2^ = 6.38, *p* < 0.1; 3 s, χ^2^ = 7.12, *p* < 0.1; 3.5 s, χ^2^ = 4.63, *p* = 0.201, identical to both the classification accuracy and ITR). For all window sizes, PRS-Square showed the highest performance in terms of both classification accuracy and ITR, although statistically significant differences were not observed.

**Figure 10:**
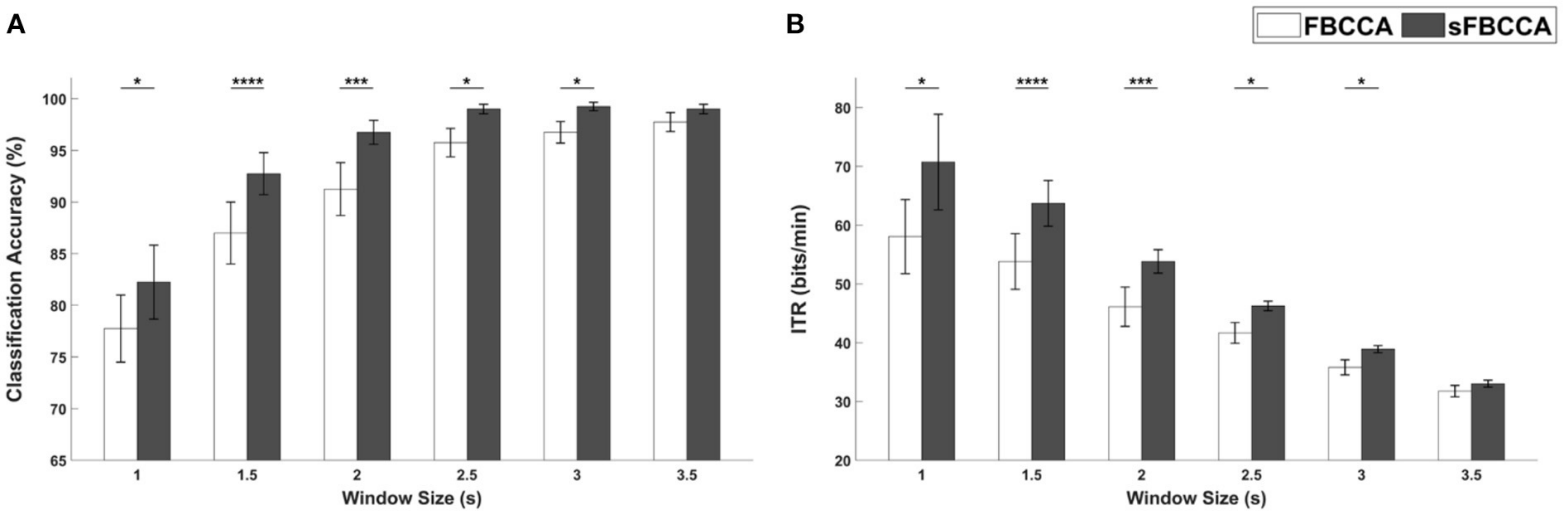
**(A)** Classification accuracies and **(B)** ITRs of SSVEP-based BCI for PRS-Square evaluated using FBCCA and sFBCCA with different window sizes. Error bars represent the standard errors. Here, *, ***, and **** represent *p* < 0.05, *p* < 0.005, and *p* < 0.001, respectively (Wilcoxon signed-rank test).

To investigate the effect of sFBCCA, the SSVEP-based BCI performances for PRS-Square were evaluated using FBCCA and sFBCCA with respect to different window sizes. In Figure 10, the white and gray bars indicate the averaged classification accuracies and ITRs evaluated using FBCCA and sFBCCA, respectively. The error bars represent standard errors. The SSVEP-based BCI performances evaluated using sFBCCA were significantly improved compared to those evaluated using FBCCA for every window size except 3.5 s (Wilcoxon signed-rank test). The result demonstrated that the proposed sFBCCA could significantly improve the performance of SSVEP-based BCIs when PRS-based hybrid visual stimuli are employed.

The fatigue scores for PRS-based hybrid visual stimuli are illustrated in Figure 6B as a function of periodic motion waveforms. The gray bars represent the interquartile ranges from 25 to 75% and white circles indicate the median values. For PRS-None, PRS-Square, PRS-Triangular, and PRS-Sine, the averaged fatigue scores were reported as 3.85 ± 1.63, 4.05 ± 1.28, 4.55 ± 1.43, and 4.8 ± 1.51, respectively.

### Comparison between FS-sine and PRS-square

**Figure 11:**
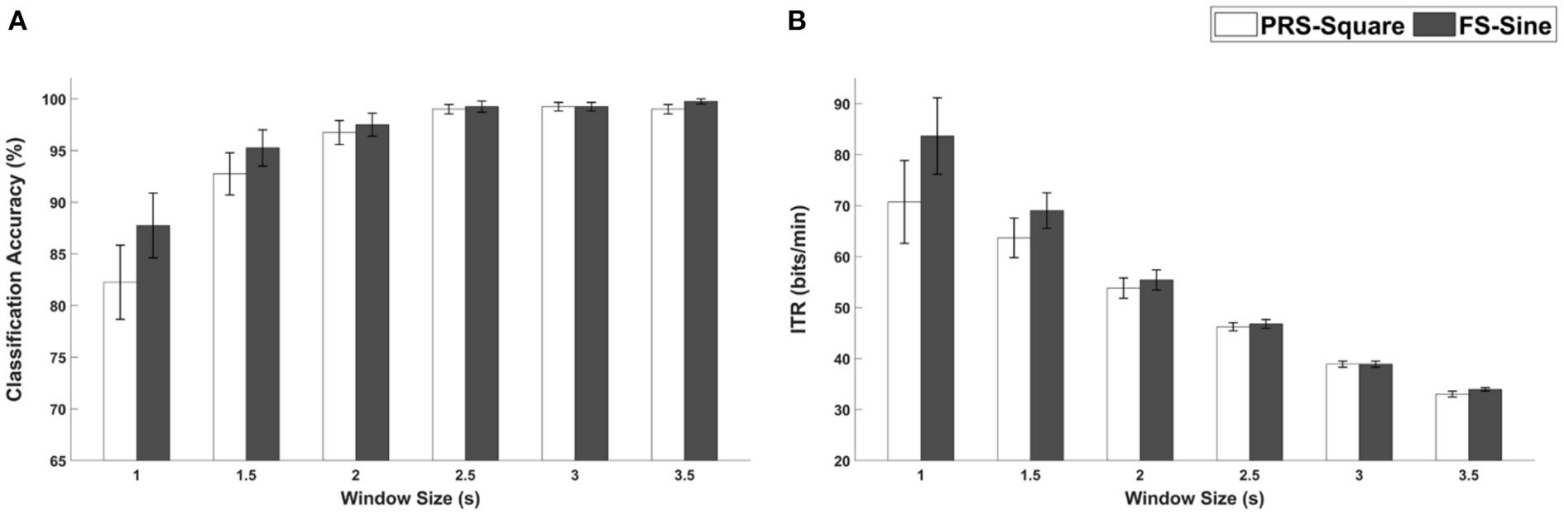
**(A)** Classification accuracies and **(B)** ITRs for PRS-Square and FS-Sine with different window sizes. Error bars represent the standard errors.

The average classification accuracies and ITRs for FS-Sine and PRS-Square, which exhibited the highest performances among FS- and PRS-based hybrid visual stimuli, are shown in Figures 11A,B, respectively. The differences in the average classification accuracies were reported to be 5.5, 2.5, 0.75, 0.25, 0.00, and 0.75%p for window sizes of 1, 1.5, 2, 2.5, 3, and 3.5 s, respectively. As for the ITRs, the differences were 12.93, 5.35, 1.60, 0.56, 0.00, 0.94 bits/min for the 1-, 1.5-, 2-, 2.5-, 3-, and 3.5-s window sizes. Statistically significant differences were not observed (Wilcoxon signed-rank test).

**Figure 12:**
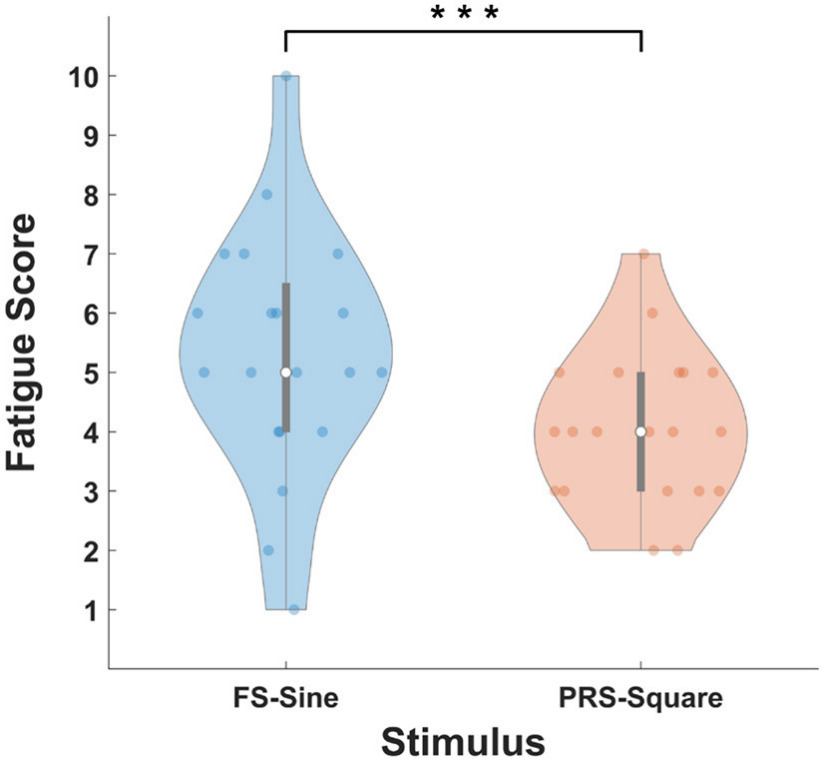
Average fatigue scores for FS-Sine and PRS-Square. Gray bars represent the interquartile range from 25 to 75% and white circles indicate the median values. Here, the asterisk of *** represents *p* < 0.005 (Wilcoxon signed-rank test).

The violin plot in Figure 12 illustrates the fatigue scores for FS-Sine and PRS-Square. The distributions of fatigue scores from the first quartile to the third quartile are presented as gray bars and the median values are indicated as white circles. The average fatigue scores were reported to be 5.3 ± 2.05 and 4.05 ± 1.28 for FS-Sine and PRS-Square, respectively. A statistically significant difference in the fatigue score was observed between the FS-Sine and PRS-Square conditions (*p* < 0.005, Wilcoxon signed-rank test), implying that PRS-Square is more visually comfortable to the users than FS-Sine.

## Discussion

In this study, we proposed novel types of hybrid visual stimuli that incorporate periodic motions into conventional SSVEP visual stimuli. Periodic motions were realized by changing the size of the visual stimulus according to four different types of waveforms. We then investigated the effect of periodic motion waveforms for the hybrid visual stimuli on the performances of SSVP-based BCIs, for the first time. Our results demonstrated that the conventional SSVEP visual stimuli combined with appropriate periodic motions could increase the SSVEP amplitudes significantly, resulting in the enhancement of SSVEP-based BCI performances. For FS, the hybrid stimulus of FS-Sine elicited the highest SSVEP amplitudes at the fundamental and second harmonic frequencies, thereby resulting in the highest average performances in terms of classification accuracies and ITRs for every window size except for 3 s. As for PRS, PRS-Square evoked the highest SSVEP components, thereby exhibiting the highest performances for all window sizes. No statistically significant difference in the performances between FS-Sine and PRS-Square was observed; however, the visual fatigue score of PRS-Square was significantly lower than that of FS-Sine. Visual fatigue is one of the main obstacles to implementing practical SSVEP-based BCIs because visual fatigue generally decreases SSVEP amplitudes, yielding degradation of overall SSVEP BCI performances (Makri et al., 2015; Ajami et al., 2018). Therefore, our results suggest that the proposed PRS-Square is the most appropriate stimulus that could improve the SSVEP-based BCI performance without inducing high visual fatigue. It is believed that the use of PRS-Square stimuli has a great potential to improve the practicality of SSVEP-based BCIs, particularly for long-term use.

We hypothesized that the performances of SSVEP-based BCIs with any kind of hybrid visual stimuli could outperform those with conventional SSVEP visual stimuli because the hybrid visual stimuli could induce both SSVEP and SSMVEP. However, unlike our expectation, FS-Square and PRS-Triangular exhibited lower average classification accuracies and ITRs than the conventional visual stimuli for some window sizes. In addition, the periodic motion of the same waveform showed different effects on FS and PRS. For example, contrary to FS-Square, PRS-Square achieved the highest classification accuracies and ITRs for every window size, suggesting that it is important to combine conventional visual stimuli with periodic motions with appropriate waveforms for implementing high-performance SSVEP-based BCIs. We believe that these results might originate from different mechanisms of FS and PRS to evoke SSVEP. However, imaging modalities with higher spatial resolution such as fMRI would be necessary to further investigate the mechanisms of FS and PRS to evoke SSVEP. On the other hand, the luminance or pattern of FS and PRS was changed according to the square waveform because previous studies (Teng et al., 2011; Chen et al., 2019) demonstrated that square-wave FS achieved significantly higher classification performances than other waveform stimuli. However, as there is a possibility that the hybrid visual stimuli combining FS or PRS of other waveforms with periodic motions might improve the performance of SSVEP-based BCI, further investigations would be necessary for the future.

**Figure 13:**
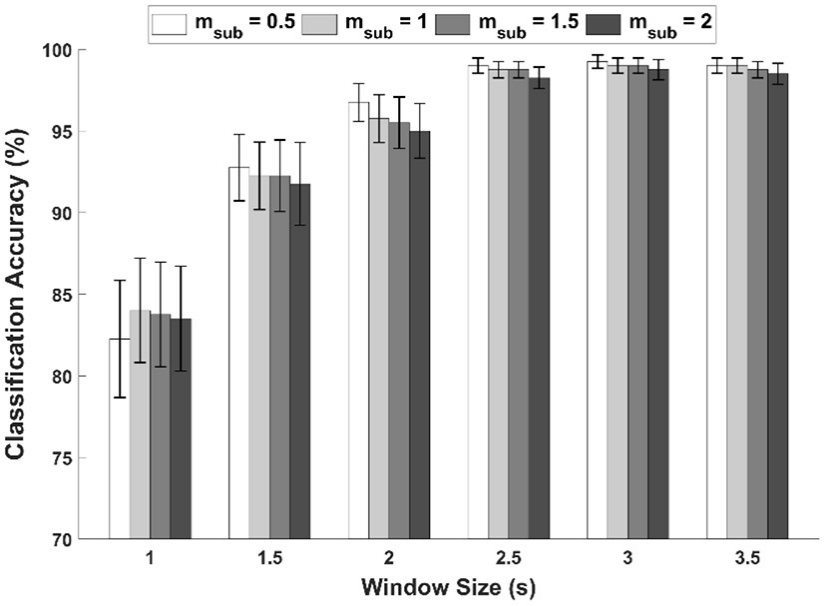
Average classification accuracies for PRS-Square as a function of *m*_*sub*_ in sFBCCA with different window sizes.

In contrast to the FS-based hybrid visual stimuli, the PRS-based hybrid visual stimuli were implemented by incorporating periodic motions whose stimulation frequency was half of PRS frequency. Although not mentioned in this manuscript, we also tested PRS-based hybrid visual stimuli with periodic motions of which the stimulation frequency was the same as that of PRS in our preliminary tests. For PRS with periodic motions of twice the frequency, most participants complained of severe visual fatigue and discomfort due to the rapid change in the stimulus size although almost the same classification accuracy as the PRS-based hybrid visual stimuli employed in this study was reported. As a result, the stimulation frequency of periodic motions was determined as half of PRS frequency. Since the use of the reduced stimulation frequency for periodic motions evoked subharmonic component, we extended the conventional FBCCA and proposed sFBCCA to fully exploit useful information contained in SSVEP evoked by the proposed PRS-based hybrid visual stimuli. The use of sFBCCA significantly enhanced the classification performances, compared to the results of FBCCA applied to PRS-Square, as shown in Figure 10. In the sFBCCA, the index of subharmonic, *m*_*sub*_, was set to 0.5, which showed the highest classification accuracies for all window sizes except 1 s, as shown in Figure 13. However, there is still a possibility of further improvement of SSVEP-based BCI performances by optimizing sFBCCA parameters. Additionally, PRS-based hybrid visual stimulus has a promising possibility of increasing the number of commands limited by the refresh rate of the LCD monitor (Li et al., 2021), thanks to its characteristics of dual main stimulation frequencies induced by SSVEP and SSMVEP. This would be one of the promising topics we would like to further investigate in our future studies for implementing high-performance SSVEP-based BCIs.

## Data availability statement

The raw data supporting the conclusions of this article will be made available by the authors, without undue reservation.

## Ethics statement

The studies involving human participants were reviewed and approved by Institutional Review Board Committee of Hanyang University. The patients/participants provided their written informed consent to participate in this study.

## Author contributions

JK designed the experiment, developed the algorithm, and analyzed the data. JH developed the visual stimulation program. HN performed experiments. C-HI supervised the study. JK and C-HI wrote the manuscript. All authors reviewed the manuscript.

## Funding

This work was supported in part by Hyundai Motor Group in 2021. The funder was not involved in the study design, collection, analysis, interpretation of data, the writing of this article, or the decision to submit it for publication. This work was also supported in part by the National Research Foundation of Korea (NRF) grant funded by the Korea Government (MSIT) (No. NRF2019R1A2C2086593), and in part by the Institute for Information and communications Technology Promotion (IITP) grant funded by the Korea government (MSIT) (2017-0-00432).

## Conflict of interest

The authors declare that the research was conducted in the absence of any commercial or financial relationships that could be construed as a potential conflict of interest.

## Publisher's note

All claims expressed in this article are solely those of the authors and do not necessarily represent those of their affiliated organizations, or those of the publisher, the editors and the reviewers. Any product that may be evaluated in this article, or claim that may be made by its manufacturer, is not guaranteed or endorsed by the publisher.
