# Supplementary material for: Novel hybrid visual stimuli incorporating periodic motions into conventional flickering or pattern-reversal visual stimuli for steady-state visual evoked potential-based brain-computer interfaces
Source: Front Neuroinform. 2022 Sep 21;16:997068. doi: 10.3389/fninf.2022.997068 (PMC9534124; doi:10.3389/fninf.2022.997068)
Supplement: Supplementary file 1 [file Data_Sheet_1.pdf]

Supplementary Material

Supplementary Table 1.

The grand mean SSVEP amplitudes at subharmonic, fundamental, and second harmonic frequencies for FS-based hybrid visual stimuli

FS		Periodic Motion Waveform			
		None	Square	Triangular	Sine
Subharmonic Component	6 Hz	0.352	0.534	0.654	0.463
	6.67 Hz	0.500	0.491	0.432	0.465
	7.5 Hz	0.555	0.430	0.464	0.513
	10 Hz	0.489	0.336	0.341	0.289
	Mean	0.474	0.448	0.473	0.433
Fundamental Component	6 Hz	0.988	1.509	1.514	1.602
	6.67 Hz	1.341	1.487	1.778	1.636
	7.5 Hz	1.697	1.687	2.334	2.496
	10 Hz	1.938	2.099	2.310	2.631
	Mean	1.491	1.696	1.984	2.091
Second Harmonic Component	6 Hz	1.611	1.316	1.726	2.398
	6.67 Hz	1.846	1.352	1.913	2.150
	7.5 Hz	1.481	1.263	1.591	1.827
	10 Hz	1.117	0.878	0.934	1.046
	Mean	1.514	1.202	1.541	1.855

Supplementary Table 2.

The grand mean SSVEP amplitudes at subharmonic, fundamental, and second harmonic frequencies for PRS-based hybrid visual stimuli

PRS		Periodic Motion Waveform			
		None	Square	Triangular	Sine
Subharmonic Component	6 Hz	0.559	1.928	1.356	1.561
	6.67 Hz	0.608	1.708	1.416	1.713
	7.5 Hz	0.460	2.033	1.480	1.962
	10 Hz	0.348	1.827	1.442	1.812
	Mean	0.494	1.874	1.424	1.767
Fundamental Frequency	6 Hz	1.721	1.920	1.318	1.517
	6.67 Hz	1.669	1.773	1.475	1.595
	7.5 Hz	1.577	1.991	1.424	1.679
	10 Hz	1.796	1.745	1.560	1.750
	Mean	1.691	1.857	1.444	1.635
Second Harmonic Component	6 Hz	1.005	1.159	0.825	0.860
	6.67 Hz	1.146	1.003	0.934	0.820
	7.5 Hz	0.902	1.017	0.768	0.803
	10 Hz	0.748	0.642	0.511	0.710
	Mean	0.950	0.955	0.760	0.798
